# Uterine artery doppler and pregnancy-associated plasma protein-A in pregnancies with fibroids

**DOI:** 10.1016/j.clinsp.2025.100615

**Published:** 2025-03-13

**Authors:** Lida Anwari

**Affiliations:** EGA Institute for Women's Health, University College London, London, UK

**Keywords:** Pregnancy outcome, Pregnancy-associated plasma protein-a, Uterine artery doppler, Fetal weight, Preeclampsia, Low birth weight infant

## Abstract

**Background:**

Uterine fibroids affect maternal and neonatal outcomes adversely. Pregnancy-Associated Plasma Protein-A (PAPP-A) and Uterine Artery Doppler (UAD) are used in the first and second trimesters to predict maternal and neonatal outcomes, including maternal preeclampsia, Small-for-Gestational-Age (SGA), and Low Birth Weight (LBW) babies.

**Methods:**

A retrospective review of medical records over 8-months was carried out for 60-patients who presented to the antenatal outpatient clinic. Inclusion criteria were the identification of fibroids in the first-trimester scan, PAPP-A blood test performed at the first visit, and UAD recorded at 20-weeks scan. Demographic characteristics, clinical parameters, pregnancy-related complications, and obstetric outcomes were extracted for data collection. Data analysis was performed to determine correlations between UAD parameters and PAPP-A levels and fibroid measurements for different fibroid types, and to determine the effect of fibroid presence on actual and estimated fibroid weight.

**Findings:**

Of 60 included patients, the mean age of patients was 35.8 ± 4.8 years, and each pregnant woman had an average of 2.9 ± 1.7 fibroids, with the majority (73 %) being large (> 5 cm). No complications, such as gestational hypertension, gestational diabetes, preeclampsia, abruption, and preterm birth were reported; only 6 (10 %) of women were sonographically diagnosed with SGA babies, while 2 (3.3 %) babies were LBW. UAD parameters and PAPP-A levels had no significant association with fibroid size (*p* > 0.05).

**Conclusion:**

There is a possible role of UAD and PAPP-A in determining pregnancy outcomes in the presence of fibroids, which needs to be explored by prospective studies.

## Highlights

• Uterine fibroids adversely impact maternal and neonatal outcomes.
• UAD parameters and PAPP-A levels do not markedly associate with the fibroid size.
• UAD and PAPP-A play functional roles in determining the outcomes of pregnancies carrying fibroids.

**Statement of Significance**

**Problem or Issue**

Uterine artery doppler and pregnancy-associated plasma protein-A are two parameters that help determine the level of risk associated with some obstetric outcomes, including fetal growth restriction and preeclampsia. However, the relation of these factors to fibroids in pregnancy is poorly understood.

**What is Already Known**

In pregnant women with uterine fibroids, while pregnancy routinely progresses in the case of small fibroids (< 5 cm), larger fibroids (> 5 cm) can primarily increase the risk for adverse pregnancy outcomes, in particular malpresentation, postpartum hemorrhage. Pregnancies associated with larger-sized uterine fibroids are at high risk of either miscarriage or pre-maturity and having a caesarean section.

**What This Paper Adds**

Selected pregnant women with large uterine fibroids had no pregnancy-related complications, including gestational hypertension and diabetes, preeclampsia, abruption, and preterm birth. Variations in the parameters significant for determining the level of risk for obstetric outcomes, including uterine artery doppler and pregnancy-associated plasma protein-A are neither correlated with the size extension of uterine fibroids nor with other adverse obstetric outcomes. Timely management of larger-sized fibroids led by the parameters predicting obstetric outcomes can prevent life-threatening postpartum hemorrhage and adverse maternal cardiovascular and neonatal outcomes.

Alt-text: Unlabelled box

## Introduction

Uterine fibroids are the most common benign tumors in pregnant women. These structures are of different geometries and sizes (commonly from 1 to 35 cm); they are composed of smooth muscle tissue and can grow both in the uterine wall and cavity and outside the uterus. Several fibroids commonly appear in the uterine muscle simultaneously.^1^ *Intramural* myoma is the most common type and is located in the smooth muscle wall of the uterus (myometrium).^2^ A myoma that grows inward into the uterus lining is called a *submucosal* myoma. These fibroids are less common, usually smaller, and cause bleeding disorders.^2^ *Subserous/subserosal* fibroids are located outside the uterine wall and grow from the muscle layer of the uterus outward into the peritoneum. This type of myoma does not cause menstrual disorders but can exert pressure on neighboring organs and structures, grow stalked, and lead to pain and complications through stem rotations.^3^ A myoma in the connective tissue on the side of the uterus is *Intra-ligamentary*, and, finally, those in the cervix are called *cervical* myomas.

The causes for the formation of uterine fibroids in women are so far unknown. One possible assumption is that there is an association with the female sex hormone estrogen, which among other controlling factors, including race and genetic predisposition, is responsible for the growth of the uterine lining (endometrium) and plays a role in the development of the muscular layer in the uterine wall.^4^ A piece of evidence that can strengthen the present assumption is that myomas/fibroids are primarily diagnosed in the uteruses of premenopausal women who mainly suffer from abnormal bleeding.^5^ It is broadly accepted that the medications with direct effects on the normal release of oestrogen (e.g., ampicillin), chronic diseases, including diabetes mellitus and disorders of the liver, kidney, thyroid gland, and adrenal glands, and other medical complications with recognized effects on the production and metabolism of estrogen (e.g., liver disease and obesity) can increase the chance of uterine fibroid formation associated with bleeding disorders in women.^6^ Accordingly, with the decrease in estrogen production and the onset of menopause, no new fibroids develop, and existing fibroids either regress or are no longer symptomatic.^7^ Fibroids occur in 20 % to 30 % of women over 30 and almost 40 % of women older than 50.^8^ Over 70 % of all women generally develop fibroids before menopause.^8^ At least 25 % of all women have ailments and symptoms of uterine fibroids and must undergo treatment.

Another theory for the cause of myoma/fibroid formation links to a genetic predisposition, i.e., an increased probability of developing a specific disease due to gene mutations or a family history that represents a higher risk of the disease.^9^ For instance, fibroids occur much more frequently in black African and American women than Caucasian women.^10^ The influence of demographics has been seen in several studies by,^11^, ^12^, ^13^, ^14^ reported in a large retrospective study of 109,000 Chinese women during pregnancy a prevalence of 2.68 % for fibroids, whereas 16.7 % of women had fibroids in Cameroon, as reported by^11^^,^^12^^,^^14^ reported in their studies prevalence values of 8.2 % and 3.2 % for UK and US women, respectively. Fibroids are commonly family-specific and can be related to family clusters.^15^ There is also an existing theory that the influence of growth hormones, particularly insulin-like growth factors, can accelerate fibroid growth by stimulating cell proliferation in uterine tissue.^16^ In a different theory, enrichment in the Extracellular Matrix (ECM) in fibroids leads them to develop a fibrous structure, as the ECM is the material that causes cells to stick together like mortar between bricks.^17^

According to,^18^ for a majority of cases, particularly in the case of small single fibroids (<5 cm), the pregnancy will progress without any problem. In contrast, in the case of larger-sized fibroids (>5 cm), some women may encounter adverse pregnancy outcomes, such as miscarriage, malpresentation, preterm labor, pain, placenta abruption, obstructive labor, PPH, and Caesarean Section (CS).^19^ The aforementioned adverse obstetric outcomes of cases with large fibroids have been further discussed and presented, and the occurrence of those outcomes has been predicted in other studies.^20^

According to,^13^ the consistently reported significant adverse obstetric outcomes associated with fibroids, based on their frequency of occurrence, are malpresentation (*p* < 0.001), CS (*p* < 0.001), and PPH (*p* > 0.04) (Table 1). Other adverse outcomes have been less consistent across studies^20^ (Table 1). A recent study by^21^ refuted the link between miscarriage and large fibroids.

**Table 1:** Review of literature for uterine fibroids in pregnancy.

Location and research type	Prevalence (Nº of women with and without fibroids)	Miscarriage rate/1000 pregnancies (%)	Malpresentation on (%)	Placenta Previa (%)	Preterm birth (%)	CS/ pregnancies with and without fibroids (%)	PPH ≥500 mL blood loss (%)	PAPP-A (MOM)	LBW <2500*g* (%)	UAD	References
China Retrospective cross-sectional study	3012 (2.7 %) with fibroids vs. 109,391 (97.3 %) without	–	6.9 with fibroids vs. 3.5 without *p* < 0.001	1.9 with fibroids vs. 1.2 without *p* = 0.16	9.1 with fibroids vs. 8.2 without *p* = 0.906	72.7 with fibroids vs. 39 without *p* < 0.001	6.6 with fibroids vs. 3.7 without	–	7.9 with fibroids vs. 7 without *p* = 0.966	–	^13^^]^
Cameroon Retro-cross- sectional study	38 (14.4 %) with fibroids vs. 226 (85.6 %) without	36.4 vs. 32.7 % *p* = 0.74	–	–	13.6 with fibroids vs. 9.1 without *p* = 0.5	31 with fibroids vs. 9.1 without *p* < 0.008	40.9 with fibroids vs. 12.7 without *p* = 0.003	–	13.6 with fibroids vs. 4.5 without *p* = 0.12	–	^14^
Italy Retrospective cohort study	219 with fibroids vs. 34 without	–	11.8 with fibroids vs. 2.7 without *p* = 0.04	–	29.4 with fibroids vs. 5 without *p* < 0.001	73.5 with fibroids vs. 37 without *p* < 0.001	–	–	–	–	^25^
USA Retrospective cohort study	2058 (3.1 %) with fibroids vs. 64,047 (96.9 %) without	–	5.3 with fibroids vs. 3.1 without *p* < 0.01	1.4 with fibroids vs. 0.5 without *p* < 0.01	15.1 with fibroids vs. 10.5 without *p* < 0.01	33.1 with fibroids vs. 24.2 without *p* < 0.01	–	–	13.7 with fibroids vs. 13.1 without *p* = 0.34	–	^12^
Pakistan Prospective study one year follow-up	10,842 (99.3 %) with fibroids vs. 80 (0.7 %) without	10 % with fibroids	12.5 % with fibroids	2.7 % with fibroids	10 % with fibroids	70 % with fibroids	38.7 % with fibroids	–	6.3 % with fibroids	–	^26^

LBW, Low Birth Weight.

While Uterine Artery Doppler (UAD) and Pregnancy-Associated Plasma Protein-A (PAPP-A) are considered to be two variables useful for determining the level of risk for some obstetric outcomes, such as Foetal Growth Restriction (FGR) and preeclampsia, up to present, there are no studies focused on demonstrating the association of these factors with fibroids in pregnancy. UAD assesses the blood flow of the uterine vessels,^22^ which clinicians can use to predict the possibility of the development of FGR and preeclampsia. The PAPP-A marker is assessed as part of the combined pregnancy screening blood test, with low levels sometimes associated with lower birth weight, preterm birth, preeclampsia, and mid-trimester miscarriage.^23^

Given the significance of the UAD and PAPP-A for predicting the likely adverse pregnancy outcome and the absence of former research targeting these predictive factors, the present study aims to examine the prevalence of these two markers in pregnancies with known uterine fibroids. The objectives of this work are two-fold:

- To determine whether the presence of fibroids in pregnant patients affects the levels of UAD and PAPP-A and to understand the underlying reasons for the alteration these parameters display in response to the development of uterine fibroids.
- To understand whether the mode of variations in levels of UAD and PAPP-A have implications for the outcome of pregnancies diagnosed with uterine fibroids.

Here, the approach to achieving these objectives includes the retrospective interrogation of the database for pregnant patients with uterine fibroids who presented at the University College London Hospitals NHS Foundation Trust (UCLH) from January to August 2021.

## Methods

### Study population

The data used and analyzed in this retrospective research were collected from the archived electronic records of the UCLH patients using the Electronic Patient Information Centre (EPIC) healthcare software. After interrogation of the EPIC database for 400 patients who attended the antenatal outpatient clinic dedicated to fibroid in pregnancy at the UCLH over an 8-month-period between January and August 2021, 60 patients were found eligible for this study. The main criteria for selecting these patients included a presence at the antenatal outpatient clinic because fibroid was identified in the first-trimester scan, a PAPP-A blood test was performed at the time of the first visit to the clinic, and the UAD was recorded at the 20-weeks scan. Various indices, such as patient background characteristics, clinical parameters, pregnancy-related complications, and obstetric outcomes, were extracted from this Electronic Patient Record (EPR) supplier for the selected 60 pregnant patients. The derived patient characteristics include age, ethnicity, and Body Mass Index (BMI), the clinical parameters including UAD, PAPP-A, size, number, and type of fibroids, the pregnancy complications such as Gestational Diabetes Mellitus (GDM), preeclampsia, abruption, and preterm birth, and the obstetric outcomes including still/live birth, fetal affectation (Foetal Growth Restriction/Small for Gestational Age ([FGR/SGA] diagnosed before birth), actual birth weight, mode of delivery (cesarean section versus Spontaneous Vaginal Delivery [SVD]), and PPH.

### Data extraction

All the parameters mentioned above were traced from the patient's electronic notes and serial scan reports recorded during pregnancy and from the discharge summary from the hospital. The presence of fibroid in the selected pregnant women was confirmed through the evaluation of early pregnancy scan reports. The recorded PAPP-A and UAD values were extracted from the booking blood result and the second-trimester scan report (anomaly scan). The antenatal visit documentation and summary of admission to delivery suit notes were reviewed in detail, and the data regarding the entire pregnancy, fibroid-related complications during the antepartum, intrapartum and postpartum period, and the mode of delivery, whether it was a spontaneous onset of labor or induction of labor or admission for elective cesarean section, were extracted. The sample data and fibroid birth weight were revised from the discharge summary outgoing letter to the general physician.

#### Fibroid evaluation

All fibroid measurements were performed during the first-trimester scan assessment. Sonographers and fibroid medicine doctors from the antenatal outpatient clinic, UCLH, who are fully trained and have at least three years of professional work experience in scanning procedures, conducted the examination. A GE volusion 8 or 10 sono-device was used to conduct the scan, and the Viewpoint scan reporting software was used for data reporting.

The ultrasound examination was conducted both transvaginally and transabdominally. The transabdominal sonography was used for pregnancy evaluation, and the transvaginal ultrasound for fibroid assessment. The sonographic assessment during pregnancy included routine first and second-trimester screening scans. In addition, serial growth scans at 28-, 34-, and 37-weeks of gestation were also performed. The identification of fibroids was based on the standardized ultrasonographic research criteria previously described in the literature.^24^ The discrimination between fibroids and other uterine pathologic variations was based on these established criteria; the sonographers and fellows had been trained on them.

In addition to fibroid biometric and Doppler assessments, examination of maternal structure, including review and remeasurement of fibroid and reassessment of fibroid location, most importantly, the cervical distance and its relation to the fibroid and distance to lower uterine segment were assessed during each scan evaluation. The size of each fibroid was measured along the three perpendicular planes sagittal, longitudinal, and transverse. Three diameters were recorded for each fibroid.

A standardized diagram for the uterus where each fibroid is mapped and numbered was then used so that each fibroid was mapped and measured separately for women with multiple fibroids. The fibroid was categorized by type and location and described as intramural fibroid if it grows within the myometrium, subserosal if the fibroid growth occurs toward the external uterine surface, and submucosal when the fibroid protrudes into the uterine cavity.

#### PAPP-A

As part of the first trimester assessments, the PAPP-A concentrations were measured using a thermo-scientific Brahms Kryptor gold analyzer. This Krypton instrument employs a Time-Resolved Amplified Cryptate Emission (TRACE) technology to maintain the high precision required for getting certainty on the measured PAPP-A value during pregnancy. Due to the completed immune reaction, the technology is based on non-radiating energy transfer from donor-type molecules (europium cryptate) to an acceptor molecule (XL665). A procedure for large-scale preparation of the circulating PAPP-A from the blood of pregnant patients is described in detail.^27^ The amino acid and carbohydrate compounds of the isolated and carboxymethylated PAPP-A were determined during this procedure. The measured absolute values of PAPP-A were then converted into Multiples of the Median (MOM) as a gestational age-dependent expression of PAPP-A levels, adjusting for fetal Crown Rump Length (CRL), maternal age, and maternal weight as formerly outlined in.^28^

#### UAD

As part of the second-trimester assessments (starting from week-20 of pregnancy), the UAD index was measured during a 20-week anomaly scan using the GE volusion 8 ultrasound machines placed at the UCLH antenatal outpatient clinic. The transabdominal ultrasound method of Doppler assessment was applied to obtain a midsagittal part of the cervical canal. At the level of the groin area, the UAD was identified with the help of gentle tilting of the transducer from side to side around the groin. The sonographer applied color Doppler mapping to spot the uterine arteries as aliasing vessels cross-cutting the Internal Iliac Arteries (IIA), used as a landmark for locating the uterine artery. The Pulsatility Index (PI) was drawn separately for left and right uterine arteries by taking the mean value of three sequential waveforms. A mean PI value for the left and right uterine arteries was then calculated.

#### EFW and birth weight

EFW and actual birth weight in the presence of fibroid were also examined. These data were extracted from scan reporting records performed at 37-weeks of gestation (referred to as growth scan) using viewpoint reporting software. The EFW was derived based on the Hadlock statistic,^29^ which adds the measurements of head circumference, abdomen circumference, and femur length. The EFW demonstrates a percentile range, which depends on gestational age. According to this weight range, the 3rd centile represents a very small baby while the 95th centile indicates a large baby. The EFW values for 37-weeks growth scan were extracted from the viewpoint reporting system for all patients involved in this study and plotted into a customized growth chart. This chart is specifically generated by taking into account each patient's ethnicity, BMI (height and booking weight), and age. Furthermore, the actual birth weight for each patient was derived from the postnatal discharge summary report.

## Results

During the first-trimester scan, sixty pregnant patients were diagnosed with uterine fibroids. A compilation of background features, clinical parameters, pregnancy-related complications, and obstetric outcomes derived from the EPR supplier for the 60 pregnant women is given in Table 2.

**Table 2:** Descriptive statistics on background features, clinical parameters, pregnancy-related complications, and obstetric outcomes (*n* = 60).

Characteristic	Descriptive stats
Demographics	
Age – mean (SD)	35.8 (4.8)
Ethnicity (%)	
*White*	40 (66.7 %)
*Black*	14 (23.3 %)
*Asia*	6 (10 %)
BMI – mean (SD)	25.8 (5.0)
BMI category (%)	
*Normal*	28 (46.7 %)
*Overweight*	20 (33.3 %)
*Obese*	8 (13.3 %)
*Morbid obesity*	2 (3.3 %)
Fibroids	
Number of fibroids ‒ mean (SD)	2.9 (1.7)
Only intramural	43 (71.7 %)
Only subserosal	7 (11.7 %)
Intramural and subserosal	10 (16.7 %)
Length ‒ range (min‒max) mm	131 (30 – 161)
Size (%)	
*Women with small fibroids (≤5* cm*)*	16 (26.7)
*Women with large fibroids (>5* cm*)*	44 (73.3)
UAD assessment	
Pulsatility index – mean (SD)	0.97 (0.37)
Pulsatility index – range (min‒max)	2.3 (0 – 2.3)
PAPP-A assessment (MOM)	
Mean (SD)	1.02 (0.57)
Range (min‒max)	3.6 (0.1 – 3.7)
Obstetrics outcomes	
Small gestational age ‒ n (%)	6 (10)
Still-birth – n (%)	0 (0)
Caesarean delivery – n (%)	39 (65)
Spontaneous vaginal delivery – n (%)	21 (35)
PPH – n (%)	12 (20)
Birth weight – mean (SD) gm	3180.95 (467.70)
Birth weight category (%)	
*Very low (<1.5* kg*)*	0 (0)
*Low (<2.5* kg*)*	2 (3.3)
*Normal (2.5 to 4.6* kg*)*	58 (96.7)

### Population characteristics

Patients' mean (SD) age was 35.8 (4.8) years and ranged from 27 to 56 years. Almost 47 % of the pregnant patients were older than the mean age of 35.8, 53 % younger than the average. Among the 60 patients used in this study, the pregnant women aged 33 were the most frequent, with a mode value of 6. Roughly 67 % of the total pregnant patients involved in this research were white, 23 % from different black ethnicities, and 10 % from Asia. The patient's BMI ranged from 17 to 44.5, with a mean (SD) value of 25.8 (5.0). Only 2 (3.3 %) out of the 60 pregnant women had BMIs in the underweight range (below 18.5), 28 (46.7 %) in the healthy weight range (18.5 to 24.9), 20 (33.3 %) in the overweight range (25 to 29.9), and 8 (13.3 %) in the obese range (30 to 39.9). The remaining 3.3 % with BMIs ≥ 40 were classified as having morbid obesity.

### Descriptive statistics from fibroid evaluation

Among 175 uterine fibroids, these 60 pregnant women had, 171 were distinct and used in this study. The remaining four fibroids were questionable and not used in the analysis. Of the 60 patients, 43 (71.7 %) women were diagnosed to have only intramural fibroids grown within their myometrium, and 7 (11.7 %) patients had subserosal fibroids, which occurred toward the external surface of the uterus. In contrast, no submucosal fibroids protruding into the uterine cavity were found. 10 (16.7 %) of the 60 pregnant women had both intramural and subserosal fibroids. Of a total number of 171 fibroids identified, 116 (67.8 %) were intramural, 15 (8.8 %) subserosal, and 40 (23.4 %) were a combination of intramural and subserosal.

It was observed that only 3 (5 %) pregnant women had fibroids in the cervix, while a majority of the patients (57 women, 95 %) carried corpus fibroids. There were no pedunculated fibroids in this cohort. Another observation was that regardless of the type of uterine fibroids, 16 patients (26.7 %) were diagnosed as having a single fibroid (only one fibroid in the uterus) and that the single-fibroid uteruses were the most frequent among the studied samples. While the mean (SD) number of fibroids in the pregnant uterus was 2.9 (1.7), it was noticed that only one patient had an exceptionally high nine fibroids in her uterus. Among the 171 uterine fibroids investigated, the largest size, the longitudinal length, reached 161 mm, while the smallest size, the transverse diameter, was 30 mm, suggesting a wide range of sizes (161‒30 = 131 mm). When assessed separately, overall values were between 40 and 161 mm for the measured longitudinal diameters and from 30 to 94 mm for the transverse diameters. Although the greatest diameter was measured 161 mm, the largest volume recorded was for a fibroid with a longitudinal diameter of 143 mm (93×136×143 mm = 1809 cm^3^). The fibroid with the smallest transverse length, 30 mm, also had the smallest volume, being 30×35×40 mm^3^. The average size of selected fibroids calculated based on the mean value of transverse, sagittal, and longitudinal diameters varied from 35 to 124 mm (3.5 to 12.4 cm). Close to 27 % of the pregnant patients included in this research had small-size fibroids (≤ 5 cm), but a majority of the cases (73 %) carried larger-size fibroids (> 5 cm).

### Descriptive statistics for UAD and PAPP-A assessment

The assessed UADs for the studied pregnant patients ranged in PI values from 0 to 2.3. Close to 42 % of the patients had pulsatility indexes above the total average value of 0.97, while 58 % had PIs below the average. Among the UAD concentrations derived for the pregnant women included in this work, the patients with a PI value of 0.8 were the most common and represented the mode concentration (13 out of the total 60 patients). 10 % of the patients had abnormal results with PIs of >1.45, and 90 % with normal results having PIs <1.45. The measured PAPP-A concentrations varied between 0.1 and 3.7 MOM, with the mean value being 1 MOM. The patients with a PAPP-A value of 0.7 MOM were the most frequent samples and represented the mode of PAPP-A.

### Descriptive statistics for pregnancy-related complications and obstetrics outcomes

Among the 60 pregnant women, only 6 cases (10 %) were sonographically diagnosed with small for gestational age, and 90 % (52 women) of the cases were Appropriate for Gestational Age (AGA). N No stillbirth was found among the studied cases. There were no pregnancy complications, including gestational hypertension, GDM, pre-eclampsia, abruption, and preterm birth in this study. Thirty-nine patients (65 %) had Caesarean, and 21 patients (35 %) had spontaneous vaginal birth. 10 of the 39 pregnant women (∼26 %) who gave birth by Caesarean, had urgency category 1 emergency CS because of immediate concern for the health of either mother or the baby. Of a total of 12 pregnant patients with a reported PPH, 10 women had a blood-loss range of 900 to 2300 mL following a CS birth, while the 2 women with normal vaginal delivery had a mild PPH with estimated volume ≤500 mL. Their babies weighed at birth from 2.4 to 4.6 kg (the average weight being 3.2 kg), with only two of them defined as having a birth weight <2.5 kg, i.e., LBW babies, while the other being within a healthy birth-weight range (from 2.5 to 4.6 kg, according to World Health Organization 2004). No very low birth weight (VLBW) babies, weighing <1.5 kg at birth, were observed.

### Relationship of UAD and PAPP-A with fibroid measurements for all women

**Fig. 1:**
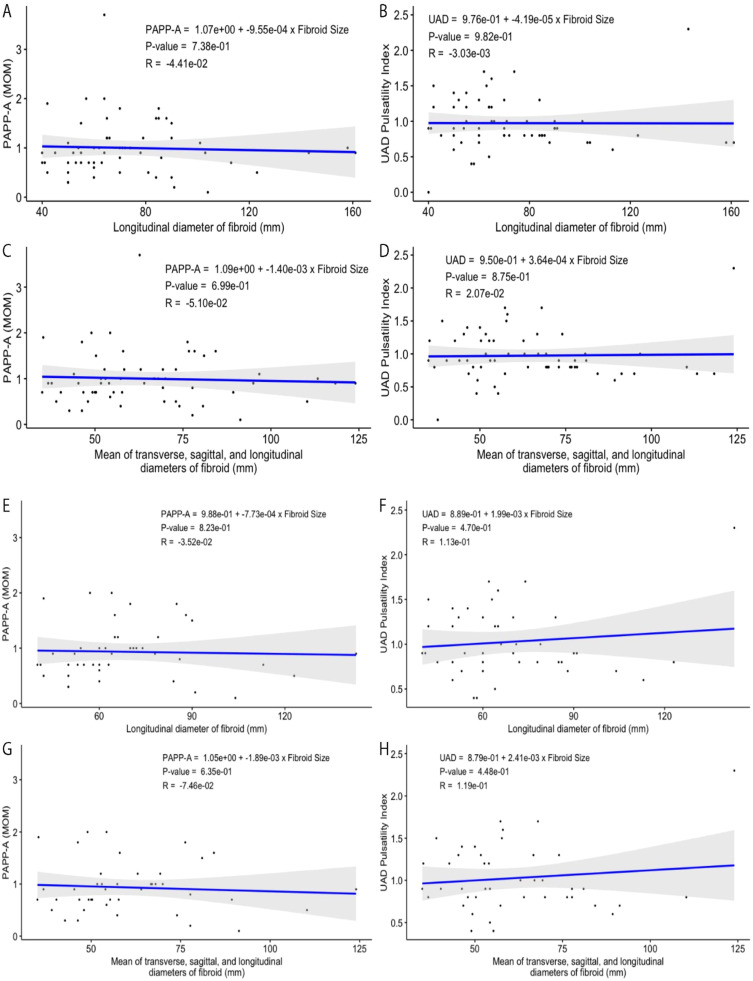
(A‒B) Relationships among fibroid longitudinal length, PAPP-A, and UAD for the selected patients diagnosed with uterine fibroids. (C‒D) Mean fibroid size (average transverse, sagittal, and longitudinal diameters) versus PAPP-A and UAD. (E‒F) Relationships among fibroid longitudinal length, PAPP-A, and UAD for the selected patients diagnosed with only intramural uterine fibroids. (G‒H) Mean intramural fibroid size (average transverse, sagittal, and longitudinal diameters) versus PAPP-A and UAD.

The PAPP-A values (MoM) plotted versus the longitudinal diameter of fibroids detected for the pregnant women involved in this study display no correlation (a very weak inverse relationship), with the value of the coefficient of determination (R^2^) being ∼0.0019 (Fig. 1A). The calculated value of Pearson correlation coefficient (*R* = −0.0441), the square root of R^2^, also shows only a weak and non-significant relationship between the Fibroid Size (FS) and PAPP-A, i.e., a negative correlation between these variables with a very low trendline slope of −0.0009. When the mean value of transverse, sagittal, and longitudinal diameters for each studied fibroid was plotted versus the PAPP-A, the negative relationship remained statistically insignificant (Fig. 1C). The longitudinal diameters of studied fibroids exhibit no correlation with the measured UAD indexes (Fig. 1B). The correlation coefficient, the coefficient of determination, trendline slope, and p-value representing the plotted fibroid size–UAD data points are −0.003, 0, 0, and 0.9818, respectively. A weak and insignificant positive correlation between the fibroid size and UAD was also observed (Figs. 1B, D).

### Relationship of UAD and PAPP-A with fibroid measurements for women with intramural fibroids

The PAPP-A values plotted versus the longitudinal diameter of fibroids detected for the pregnant women with only intramural fibroids in this study display no correlation (a very weak inverse relationship) (Fig. 1E). The calculated value of Pearson correlation coefficient (*R* = −0.0352), the square root of R^2^, also shows only a very weak relationship between the Fibroid Size (FS) and PAPP-A, i.e., a negative correlation between these variables with a very low trendline slope of −0.0008. When the mean value of transverse, sagittal, and longitudinal diameters for each studied fibroid was plotted versus the PAPP-A, the negative relationship between the fibroid size and this variable was slightly improved (Fig. 1G).

The longitudinal diameters of studied fibroids exhibit no correlation with the measured UAD indexes (Fig. 1F). The correlation coefficient, the coefficient of determination, trendline slope, and p-value representing the plotted fibroid size–UAD data points are 0.113, 0.01279, 0.00199, and 0.470, respectively, suggesting a weak positive correlation which is not statistically significant. A very weak positive correlation between the fibroid size and UAD, with the correlation and determination coefficients, trendline slope, and p-value being respectively 0.1188, 0.01411, 0.0024, 0.448, was observed for the selected samples when the longitudinal diameters were replaced with the average diameters for each fibroid (Fig. 1H). For both groups of fibroid size–UAD value data points, the calculated p-values show that the relationship between these variables is statistically insignificant for women with only intramural fibroids.

Like all women, regardless of the PAPP-A and UAD being plotted versus the longitudinal or average size of the fibroid, the fibroid size–PAPP-A data points display a rather better correlation than that of the fibroid size–UAD data points for women with intramural fibroids. Figs. 1E–H demonstrate these findings graphically.

### Relationship of UAD and PAPP-A with fibroid measurements for women with subserosal fibroids

**Fig. 2:**
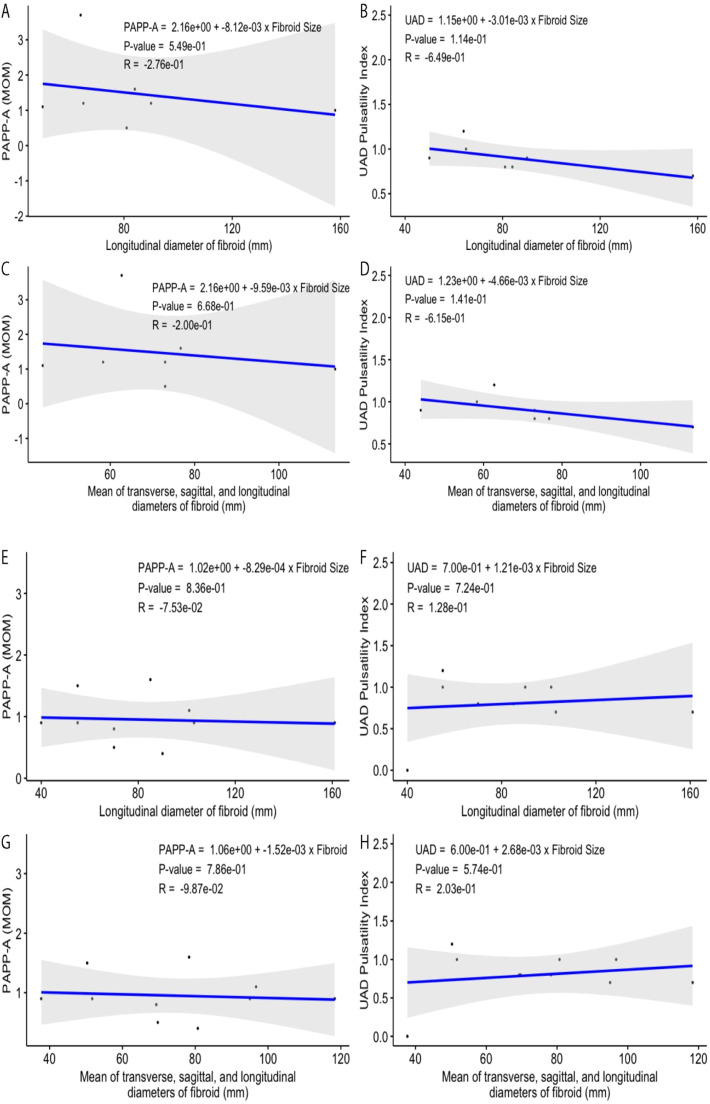
(A‒B) Relationships among fibroid longitudinal length, PAPP-A, and UAD for the selected patients diagnosed with only subserosal uterine fibroids. (C‒D) Mean fibroid size (average transverse, sagittal, and longitudinal diameters) versus PAPP-A and UAD. (E‒F) Relationships among fibroid longitudinal length, PAPP-A, and UAD for the selected patients diagnosed with intramural and subserosal uterine fibroids. (G‒H) Mean intramural-subserosal fibroid size (average transverse, sagittal, and longitudinal diameters) versus PAPP-A and UAD.

The PAPP-A values plotted versus the longitudinal diameter of fibroids detected for the pregnant women with only subserosal fibroids in this study display no correlation (a weak inverse relationship) between the data points, with the value of the coefficient of determination (R^2^) being ∼0.07622 (Fig. 2A). The calculated value of Pearson correlation coefficient (*R* = −0.27607), the square root of R^2^, also shows only a weak relationship between the Fibroid Size (FS) and PAPP-A, i.e., a negative correlation between these variables with a very low trendline slope of −0.008121. The p-value of 0.549 was calculated using the R score of −0.27607, and the number of pairs for the selected samples (7-pairs) is not statistically significant for the relationship between these variables (*p>*significance level 0.05). When the mean value of transverse, sagittal, and longitudinal diameters for each studied fibroid was plotted versus the PAPP-A, the negative relationship between the fibroid size and this variable slightly deteriorated (Fig. 2C). This scatter plot defines the correlation and determination coefficient values of respectively −0.19951 and 0.03981 with a trendline slope of −0.009587, and a p-value of 0.668 which shows the correlation between the fibroid size–PAPP-A data points is still statistically insignificant.

Similar to all women and regardless of the PAPP-A and UAD are plotted versus longitudinal or average size of the fibroid, the fibroid size–PAPP-A data points display a rather better but still weak and non-significant correlation than that of the fibroid size–UAD data points for women with intramural fibroids (Figs. 2B, D).

### Relationship of UAD and PAPP-A with fibroid measurements for women with both intramural and subserosal fibroids

The PAPP-A values plotted versus the longitudinal diameter of fibroids detected for the pregnant women with both types of fibroids in this study display no correlation (a very weak inverse relationship) between the data points, with the value of the coefficient of determination (R^2^) being ∼0.005677 (Fig. 2E). The calculated value of Parson correlation coefficient (*R* = −0.07534), the square root of R^2^, also shows only a very weak relationship between the Fibroid Size (FS) and PAPP-A, i.e., a negative correlation between these variables with a very low trendline slope of −0.00083. The p-value of 0.836 was calculated using the R score of −0.07534, and the number of pairs for the selected samples (10-pairs) is not statistically significant for the relationship between these variables (*p* > significance level 0.05). When the mean value of transverse, sagittal, and longitudinal diameters for each studied fibroid was plotted versus the PAPP-A, the negative relationship between the fibroid size and this variable was slightly improved (Fig. 2G). This scatter plot defines the correlation and determination coefficient values of respectively −0.098678 and 0.009737 with a trendline slope of −0.001523, and a p-value of 0.786 which shows the correlation between the fibroid size–PAPP-A data points is still statistically insignificant.

The longitudinal diameters of studied fibroids exhibit no significant correlation with the measured UAD indexes (Fig. 2F). The correlation coefficient, the coefficient of determination, trendline slope, and p-value representing the plotted fibroid size–UAD data points are 0.1281, 0.01642, 0.001206, and 0.724, respectively, suggesting a weak positive correlation which is not statistically significant. A weak positive correlation between the fibroid size and UAD, with the correlation and determination coefficients, trendline slope, and p-value being respectively 0.20294, 0.04118, 0.002679, 0.574, was observed for the selected samples when the longitudinal diameters were replaced with the average diameters for each fibroid (Fig. 2H).

For both groups of fibroid size–UAD value data points (Figs. 2E–H), the calculated p-values show that the relationship between these variables is statistically insignificant for women with both intramural and subserosal fibroids. Similar to all women, and regardless of the PAPP-A and UAD are plotted versus the longitudinal or average size of the fibroid, the fibroid size–PAPP-A data points display a rather better correlation than that of the fibroid size–UAD data points for women with both intramural and subserosal fibroids. Figs. 2E–H demonstrate these findings graphically.

### Relationship between estimated fetal weight versus the actual weight of the baby

**Fig. 3:**
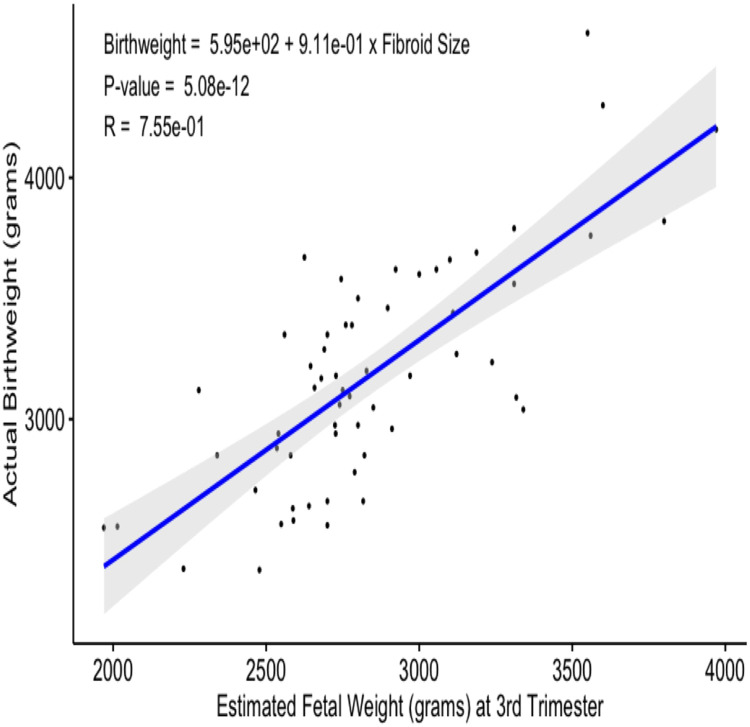
Relationship between estimated foetal weight versus the actual weight of the baby.

The mean (SD) actual weight of babies delivered at birth was 3181 grams, ranging from 2375 to 4600 grams. Estimated Foetal Weight (EFW) in the 3rd trimester was available for 59 women in the study, and the mean (SD) EFW in the 3rd trimester was 2838 grams, ranging from 1970 to 3970 grams. The actual birthweight values plotted versus the EFW in the third trimester display a strong correlation (a strong positive relationship) between the data points, with the value of the coefficient of determination (R^2^) being 0.5695 (Fig. 3). The calculated value of Pearson correlation coefficient (*R* = 0.75468) also shows a strong relationship between the EFW at third trimester and actual birthweight, i.e., a positive correlation between these variables with a high trendline slope of 0.9109. The p-value of 5.08e-12 was calculated using the R score of 0.75468, and the number of pairs for the selected samples (59-pairs) is statistically significant for the relationship between these variables (*p*< significance level 0.05). Fig. 3 demonstrates these findings graphically.

## Discussion

### Prevalence and risk factors for uterine fibroids in pregnancy

There is a high prevalence of uterine fibroids, the most common benign tumors of the female reproductive tract, in non-pregnant females, amounting from 20 %‒40 %. In pregnancy, however, the estimated incidence of fibroids is 0.1 % to 3.9 %, which can be attributed to the higher rate of infertility, lower implantation rates after In Vitro Fertilisation (IVF), and undiagnosed asymptomatic fibroids. A prevalence as high as 17 % in pregnancy has also been reported in a cohort of African women, especially in older females with low parity.^14^

The prevalence of fibroids in pregnancy is purportedly affected by racial and genetic factors.^14^ Detection of fibroids is usually carried out through ultrasonography in pregnancy; however, size threshold and trimester of pregnancy are factors that can affect detection, and with ultrasound being an operator-dependent modality, small-sized fibroids can be missed during early pregnancy as well.^18^^,^^30^ Ethnic differences in the risk of fibroid development have been reported due to increased expression of the estrogen receptors ERalpha PP-genotype, with black women having a higher prevalence compared to white and Hispanic women.^31^ The risk of fibroid development is three times higher in African American females compared to white women, and the age of onset in this ethnic cohort is also earlier by 10‒15-years.^32^

In this study, the majority of patients (67 %) were from a white ethnic background, with only 23 % from a black ethnic background, and 10 % Asians. These statistics correspond to the ethnic distribution in central London, where almost 60 % of the population is White British or Other White.^33^ Possibly due to the small size in this work, ethnicity was not significantly correlated with fibroid presentation during pregnancy. The prevalence of fibroids in pregnancy is purportedly affected by racial and genetic factors.^14^ Detection of fibroids is usually carried out through ultrasonography in pregnancy; however, size threshold and trimester of pregnancy are factors that can affect detection, and with ultrasound being an operator-dependent modality, small sized fibroids can be missed during early pregnancy as well.^18^^,^^30^ Ethnic differences in the risk of fibroid development have been reported due to increased expression of the estrogen receptors ERalpha PP-genotype, with black women having a higher prevalence compared to white and Hispanic women.^31^ The risk of fibroid development is three times higher in African American females compared to white women, and the age of onset in this ethnic cohort is also earlier by 10‒15-years.^32^

The mean age of participants in this study was 35.8 ± 4.8 years: this age of presentation is consistent with other studies, which have shown that age > 35 can be an independent risk factor for fibroid development during pregnancy.^34^, ^35^, ^36^ Although fibroids get more common with age, their risk of development during the reproductive age is almost 35 %‒77 %^14^^,^^34^ also found a significant association between older age (30‒35-years) and fibroid development during pregnancy. Nulliparity^34^ or primiparity^14^ have also been found to be significant risk factors for fibroid development in pregnancy; however, data on parity was not collected during the present study.

In this study, almost 50 % of women were in the overweight, obese, or morbidly obese category. Increased estrogen levels in overweight and obese women have been linked with an increased risk of fibroid development; other possible mechanisms of fibroid development in this population include altered sex hormone metabolism, reduced production of sex-hormone binding globulin, and systemic inflammation due to obesity.^37^ The interrelationship between BMI and fibroid development is complex: while some studies have shown a positive or inverse J-shaped correlation between them,^15^^,^^38^^,^^39^ other studies have focused more on weight gain since adulthood instead of total BMI, which has been found to be more positively associated with a risk of fibroid development,^40^ especially among parous women.^38^

### Clinical features and outcomes of fibroids in pregnancy

Most of the fibroids in this study were located either intra-murally (71.7 %) or in a sub-serous location (11.7 %), or both (16.7 %), with 95 % involving the corpus of the uterus. This localization during pregnancy has also been reported elsewhere.^14^ It correlates well with the most common symptoms with which fibroids can present during pregnancy, i.e., the feeling of pressure, pain (due to ischemia of the growing fibroid during pregnancy), increased urinary frequency (due to pressure on the bladder), and vaginal bleeding.^14^^,^^41^^,^^42^ This work did not record data on initial presentation with fibroids due to the retrospective nature and inclusion of women with diagnosed fibroids in the first trimester only.

Large-sized fibroids (>5 cm) have an increased likelihood of interfering with placentation and uteroplacental blood flow and can cause clinical risks to the mother and fetus such as IUGR, placental abruption, and preeclampsia.^18^ In this study, 73 % of the cases had large-sized fibroids, with the average fibroid size ranging from 3.5 to 12.4 cm. Large fibroids have also been linked with premature rupture of membranes and preterm birth.^25^ The number of fibroids can also influence obstetric outcomes: compared to women with no fibroids,^25^ found a higher risk for preterm birth, cesarean section, and breech presentation in women with multiple fibroids. The present work's average number of fibroids was 2.9 ± 1.7 per woman, with 26.7 % of women having a single fibroid only.

Adverse clinical outcomes associated with fibroids in pregnancy can be either maternal or neonatal. Maternal outcomes include an increased rate of delivery by Caesarean section, preterm delivery at <37-weeks, and excessive PPH.^14^^,^^43^^,^^44^ In this study, 65 % of the patients underwent Caesarean delivery, with 16.7 % encountering severe PPH after C-section (blood loss 900‒2300 mL); however, no preterm births were recorded in this study.

Neonatal adverse outcomes with pregnancy-associated fibroids can include breech presentation, intrauterine fetal death, intrauterine fetal growth restriction (IUGR), LBW babies <2500*g* , and low APGAR scores after birth.^14^^,^^44^ A large retrospective review of obstetric and perinatal outcomes, however, showed only lower gestational age and mean birthweight as significant outcomes^45^; these are similar findings to the present study, as the authors report only 2 LBW babies, with average birthweight being 3200*g*. Since data about APGAR scores at 1 and 5 min after birth was not included in the present study, this can be a potential limitation, especially since^14^ demonstrated significantly low 5-min APGAR scores (≤7) in their study in 31.8 % of the neonates. This study did not find any cases of placental abruption, premature rupture of membranes, IUGR, or breech presentation, which have been highlighted previously in other studies.^13^^,^^44^

### Utility of UAD during pregnancy

UAD is a diagnostic modality that detects uterine artery blood flow and any associated vascular abnormalities during pregnancy.^46^ Detecting changes in uterine and placental blood vessels during pregnancy, at any stage from implantation to the end, can favorably predict vascular complications through the quantitative evaluation of various blood flow indices, such as the PI, Resistance Index (RI), Systolic (S)/Diastolic (D) Ratio (SDR), Peak Systolic Velocity (PSV), End-Diastolic Velocity (EDV) and appearance of an early diastolic notch, among others.^47^^,^^48^ While UAD can be determined by both transabdominal and transvaginal ultrasonography, a comparative study done to determine uterine artery PI as a screening measure for preeclampsia found higher mean PI values with transvaginal UAD.^49^ However, the transabdominal approach is frequently used in clinical practice due to its non-invasive nature, better tolerability, and excellent repeatability^46^; consequently, all UAD assessments were performed using the transabdominal approach in this study.

Obstetric complications, which can most commonly be determined through UAD, mostly include defects of placentation, leading to preeclampsia, Foetal Growth Restriction (FGR), and Small for Gestational Age (SGA) births. While standard UAD index measurements are carried out during the second trimester at the 20-week anomaly scan, UAD measurement during the first trimester at 11‒14 weeks has also been shown to predict preeclampsia or FGR with high specificity,^50^ but low sensitivity; consideration of other parameters such as maternal characteristics and biochemical indices can increase early-onset preeclampsia prediction to >90 %..^47^ The third trimester UAD performed between 24 and 36 weeks was also found to significantly predict adverse perinatal outcomes such as preeclampsia, early preeclampsia, and SGA.^51^ Uterine artery compliance, as measured through UAD by early diastolic notch, RI and PI, has also been linked with the incidence of maternal hypertension after delivery. Reduced uterine artery compliance during a second-trimester ultrasound with higher RI and PI values was associated with increased odds for incident hypertension in mothers 2‒7 years after their pregnancy and may represent a biomarker for maternal cardiovascular disorders.^52^ Other obstetric uses of UAD include predicting preterm delivery before 37 weeks^53^ and stillbirths.^54^

The association of UAD indices with fibroids has been reported rather infrequently in the literature. One study comparing UAD indices in fibroids with or without abnormal uterine bleeding found higher Peak-Systolic Velocity (PSV), RI, PI, and SDR values in females with fibroid uterus and bleeding^55^; these indices were positively correlated with higher mean uterine volume as well. Another study comparing UAD indices before and after uterine artery embolization for fibroid treatment found a significant reduction in PSV and EDV values, which was also related to a reduction in leiomyoma volume.^26^ In a pre-pregnancy study carried out to determine implantation rates for women with and without fibroids after in vitro fertilization, Doppler examination of uterine arteries in the fibroid group prior to oocyte retrieval showed significantly lower PI and RI values of right uterine artery and average right and left uterine arteries in women with fibroids who failed to conceive.^56^ Other studies have been conducted to determine UAD in differentiating fibroids and adenomyomas^57^ and to determine the vascularity of fibroid nodules by determining perifibroid and intrafibroid arterial indices.^58^

However, none of the aforementioned studies has examined the role of UAD indices in determining fibroid progression or obstetric outcomes in women with fibroids during pregnancy.^59^ performed a color Doppler assessment of fibroids during pregnancy to analyze the correlation of Doppler flow signal with fibroid growth over the gestational period, but they did not perform UAD or measure UAD indices. To the best of my knowledge, this study is the first such work exploring UAD indices with fibroids and obstetric outcomes. In this study, UAD was performed during the 20-weeks anomaly scan only, and serial Doppler scans were not carried out to determine any changes in UAD indices with pregnancy progression. The average value of PI as measured on UAD was 0.97, with a range from 0 to 2.3; 10 % of the patients (6) had an abnormal result with PI-values >1.45 (which is high compared to the general population? Compare and comment please). Although a weak positive relation was determined between average fibroid diameter and UAD indices, this was statistically insignificant regardless of the type of fibroid. Measuring UAD indices in women with fibroids prospectively during pregnancy would provide a better idea of their significance during fibroid pregnancy.

### Utility of PAPP-A during pregnancy

PAPP-A is a serum biomarker measured during the first trimester, which can be associated with obstetric complications such as stillbirth, IUGR, preterm birth, preeclampsia, and infant death.^60^ Typically measured during first-trimester assessments at 11‒13 weeks, lower levels of PAPP-A have been linked with a high risk of adverse fetal complications, especially preterm birth, as well as increased incidence of maternal hypertensive disorders such as preeclampsia during pregnancy.^61^^,^^62^ Other studies have reported a significantly higher risk of SGA and LBW babies in women with low PAPP- A levels (≤5th percentile) during first-trimester aneuploidy screening.^63^

PAPP-A is a metalloproteinase produced by placental syncytiotrophoblasts, which interact with insulin-like growth factors (IGFs) to promote the growth of the placenta and fetus.^64^ Lower levels of PAPP-A during the first trimester are related to placental dysfunction and account for fetal metabolic disturbances leading to SGA babies.^65^ The link between PAPP-A levels and maternal complications is slightly more complex. During the first trimester, low levels of PAPP-A can predict the development of early preeclampsia. However, they require a correlation between maternal characteristics and UAD; in the second trimester, however, an even more pronounced decrement in PAPP-A levels has been noted in women who ultimately develop preeclampsia^65^^,^^66^ also found an association of low PAPP-A levels during the first trimester with long-term maternal metabolic outcomes such as the development of de novo diabetes mellitus and increased use of hypoglycaemic agents at 7 and 10-years post pregnancy. Lower PAPP-A levels can also be influenced by maternal factors, such as smoking, which largely impacts PAPP-A levels during the second and third trimesters.^67^

### PAPP-A levels in relation to fibroids during pregnancy

Only recently has research been conducted to explore the association of maternal PAPP-A levels with fibroids.^68^ conducted a retrospective study assessing 198 women with at least one non-cavity distorting intramural fibroid with a diameter of at least 2 cm who had undergone first-trimester aneuploidy screening in a tertiary care center over nine years from 2011 to 2020. They compared these findings with a control group of women over the same duration who did not have fibroids and found significantly lower levels (in multiples of median) of PAPP-A in women with fibroids compared to those without; these levels were even lower in women with fibroid size >5.5 cm. They also determined that fibroids with a size <5.5 cm did not significantly alter first-trimester aneuploidy screening test parameters; such alterations were only observed with fibroid size >5.5 cm. This research presents landmark findings that have not been previously reported elsewhere in the literature. Previously,^69^ found no significant differences in PAPP-A concentrations in women with or without uterine leiomyomas. However, they did find the maternal beta HCG levels in the first and second trimesters to be increased in the leiomyoma group compared to controls.

In this study, the mean PAPP-A levels were 1 MOM, with a range between 0.1 and 3.7 MOM. For all fibroids, a weak inverse relationship was present between fibroid size and PAPP-A levels, which was not statistically significant. Similar findings were present when the dataset was split according to different types of fibroids: any intramural, subserosal, or both fibroid size did not show a significant correlation with PAPP-A levels, although these associations fare slightly better compared to data plots for UAD indices and fibroid size. Compared to the findings of,^68^ the authors did not report any significant association between low PAPP-A levels and fibroid size. Even though a weak inverse relationship was present, signifying that lower PAPP-A levels were associated with higher fibroid size, the small overall sample size and the small number of pairs for selected sampling groups attributed to the lack of a significant association. This research also did not compare PAPP-A levels, or proportions of women with low PAPP-A, in women with fibroids to controls without fibroids, and may have missed any significant differences that could have been present between these groups.

### Effect of fibroids on estimated and actual birth weight

This study also looked at the correlation Between Estimated (EBW) and Actual Birth Weight (ABW) in the presence of fibroids. Conflicting data have been reported on the association of fibroids with LBW: hypothetically, fibroids are thought to reduce birthweight and affect gestational age by distorting the uterine cavity and interfering with placental perfusion and fetal nutrition.^25^ Although some studies have supported this hypothesis,^25^ current evidence has emerged to the contrary.^70^

In this work, the authors found a significant correlation between the mean EBW during the third trimester (2838±390.83 grams) and the actual birth weight (3181±467.70) (Pearson *R* = 0.75468; *p* < 0.05). In the absence of a control group, the authors compared the estimated and the actual birth weight to determine whether fibroids could result in decreased birthweight and found no significant association between any size of fibroids and a decrease in actual birth weight. Only two babies in this study had a birthweight of fewer than 2500 grams, with the rest having a healthy birthweight averaging 3200 grams. Thus the present study supports the recent findings of^70^ regarding birthweight in the presence of fibroids. This work did not correlate the number of fibroids with birth weight; as^70^ reported, women with three or more fibroids have an increased likelihood of birthing lighter infants.

### Limitations

This study has some limitations. Due to the retrospective nature of data collection, certain variables, such as the clinical presentation of women with vaginal pain, bleeding, or urinary symptoms, were not recorded. Due to the small sample size, associations between fibroid size according to different fibroid types and UAD and PAPP-A levels could not be significantly determined. Even though weak correlations were found, a large, powered study would have helped determine the strength and significance of associations. The authors also did not follow serial UAD scans to determine progression in indices. Nevertheless, this study is the first to report UAD indices in relation to fibroids during pregnancy. The authors also report important data on PAPP-A levels and maternal fibroids, which have only recently been explored as correlated. This retrospective study can serve as the basis of a large prospective cohort to determine whether UAD indices can predict adverse obstetric outcomes in women with fibroids and whether PAPP-A levels in the presence of fibroids can truly provide a clinically significant prediction of obstetric outcomes.

## Conclusions

A variable incidence of fibroids in pregnant women can be affected by age, race, and BMI. While ethnic influences were negligible in this study, older age and higher BMI in most participants were linked with an increased incidence of multiple fibroids. Fibroids during pregnancy can restrict fetal growth leading to preterm and low-birth-weight babies, and contribute to a higher rate of Caesarean sections and postpartum hemorrhage. The study reflected the latter, with >60 % of women undergoing Caesarean delivery; however, the authors did not record any preterm deliveries. Due to these possible maternal and neonatal risks, fibroid evaluation and management are important during pregnancy, and this study explored a possible novel role for two common modalities for the evaluation of fetal outcomes for this purpose.

UAD indices measured during the second trimester provide important predictive information about possible adverse obstetric outcomes such as preeclampsia, SGA, and LBW births. This study is the first to explore the relationship of these indices with the presence and size of fibroids, as well as their predictive value in pregnant women with uterine fibroids. Although weak correlations were present between the PI on UAD and fibroid size in this study for all types of fibroids, these were statistically insignificant. 10 % of women with fibroids in this study had a PI value higher than the cut-off of 1.45, however, a relatively high value. Nevertheless, these findings warrant a prospective exploration of the role of UAD. Serial UAD measurements can be easily carried out in women known to have fibroids and can predict those who may need assisted delivery via Caesarean section or intensive neonatal care.

PAPP-A is another important first-trimester serum marker that can screen for both chromosomal anomalies and adverse obstetric outcomes; lower PAPP-A levels in women pregnant in the presence of large fibroids can potentially guide fibroid management to avoid these outcomes. While lower PAPP-A was better correlated with increased fibroid size for all fibroid types than UAD in this study, factors such as small sample size precluded these findings from reaching statistical significance. With recent novel data emerging only recently about PAPP-A and gravid uterine fibroids, the present study provides a framework for further research in this link. Measurement of PAPP-A levels can provide a useful adjunct to other diagnostic modalities such as uterine ultrasound and can be an early screening marker for pregnancy outcomes in the presence of fibroids. Modifiable factors affecting PAPP-A levels, such as smoking, can also be measured in prospective studies; with a potential link between low PAPP-A and increased risk for maternal diabetes, the effect of fibroids in accelerating this risk can also be determined by long cohort studies.

In terms of birth weight, the authors found a strong correlation between weight estimated through pregnancy scans and actual birth weight even in the presence of large fibroids in the majority of women. While number of fibroids was not correlated with birth weight in this study, the average number was almost 3 fibroids per woman; lower birth weights have been reported mostly if fibroids exceed this average. Low PAPP-A levels in pregnancy can also be linked with decreased birth weight; considering that average PAPP-A levels in this study were near normal, this correlation was not significant. The stringent inclusion criteria this study evaluated precluded some cases, which could have added value to the measured correlations. Since both UAD and PAPP-A are non or minimally invasive and are already used in anomaly scans and first-trimester aneuploidy screening respectively, their additional utility in predicting pregnancy outcomes in women with fibroids can potentially limit maternal morbidity and be less time and cost-consuming as well. Early management of large fibroids guided by these modalities can prevent life-threatening PPH and adverse maternal cardiovascular outcomes as well as neonatal outcomes. Given the dearth of literature on this role, the present work can provide a landmark for prospective cohort studies to gauge the utility of these modalities.

## Funding

This work is not funded by any organization.

## Ethical statement

Ethical approval and registration codes are not applicable to this work.

## Data availability

The data supporting the findings of this study are available within the article.

## CRediT authorship contribution statement

**Lida Anwari:** Conceptualization, Data curation, Formal analysis, Investigation, Methodology, Resources, Validation, Visualization, Writing – original draft, Writing – review & editing.

## Declaration of competing interest

The authors declare no conflicts of interest.
